# Novel approaches for immune reconstitution and adaptive immune modeling with human pluripotent stem cells

**DOI:** 10.1186/1741-7015-9-51

**Published:** 2011-05-10

**Authors:** Michael D Green, Hans-Willem Snoeck

**Affiliations:** 1 Department of Oncological Sciences and Black Family Stem Cell Institute, Mount Sinai School of Medicine, New York, NY 10029, USA

## Abstract

Pluripotent stem cells have the capacity to generate all cell lineages, and substantial progress has been made in realizing this potential. One fascinating but as yet unrealized possibility is the differentiation of pluripotent stem cells into thymic epithelial cells. The thymus is a primary lymphoid organ essential for naïve T-cell generation. T cells play an important role in adaptive immunity, and their loss or dysfunction underlies in a wide range of autoimmune and infectious diseases. T cells are generated and selected through interaction with thymic epithelial cells, the functionally essential element of thymus. The ability to generate functional thymic epithelial cells from pluripotent stem cells would have applications in modeling human immune responses in mice, in tissue transplantation, and in modulating autoimmune and infectious disease.

## Introduction

Embryonic stem (ES) cells are derived from the inner cell mass of the blastocyst and can be maintained in a pluripotent state in defined conditions in both human and mouse [1]. Theoretically, ES cells can differentiate into every somatic and germ cell type. The recent discovery that adult somatic cells can be reprogrammed into a pluripotent state (induced pluripotent stem (iPS) cells) [2,3] opens the way for the generation of patient-specific PS cells, which would overcome rejection problems associated with transplantation of ES cell-derived tissues. The development of appropriate conditions to differentiate ES and iPS cells into a variety of cell types and tissues therefore holds major promise for future cell-replacement therapy. A major challenge in this field is the directed differentiation of pluripotent cells into functional mature tissue [1,4].

A relatively underinvestigated area is the generation of cells of the thymus from ES and iPS cells. The thymus is the site of development of T cells, cells essential for adaptive immunity. In the thymus, a self-tolerant T-cell repertoire is established through positive and negative selection [5,6]. Very early hematopoietic precursors seed the thymus from the bone marrow to initiate T cell development [7]. Hence, the thymus consists of a hematopoietic, an epithelial and a mesenchymal component. The hematopoietic component includes developing T cells and mature dendritic cells [5-7]. The epithelial component includes thymic epithelial cells (TECs), which have two subtypes: cortical (cTEC) and medullary (mTEC). These two types of TECs form a three-dimensional structure together with endothelial cells, poorly defined mesenchymal cells, neural elements, neural crest-derived pericytes, and adipocytes [5,6]. Only TECs seem to be essential, as purified undifferentiated TECs at embryonic day (E) 12.5 can reconstitute a functional thymus after aggregation and transplantation under the kidney capsule [8-11]. During T-cell maturation, early T-cell precursors enter the thymus and undergo expansion, followed by cTEC-dependent positive selection for high antigen affinity, and then mTEC- and dendritic cell-dependent negative selection against strong autoantigen recognition [12].

## Discussion

### Rationale for the generation of TECS from pluripotent stem cells

**Figure 1:**
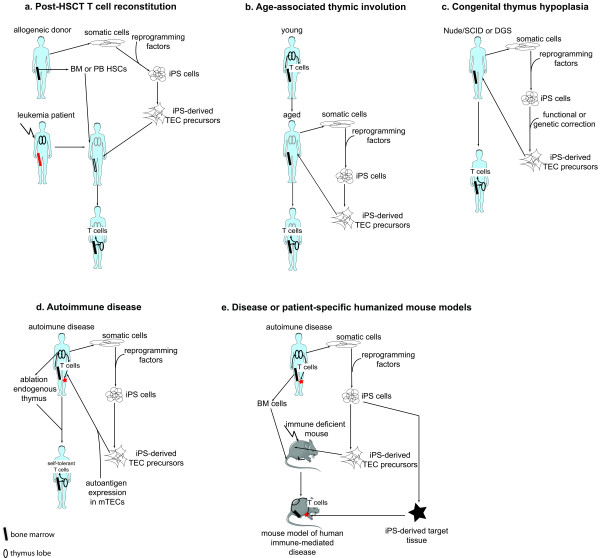
**Schematic representation of potential applications of human induced pluripotent stem (iPS) cells-derived thymic epithelial cells (TECs)**. Gray compartments = decreased cell number and function; black cellular compartments = normal cell number and function.

There are several reasons to pursue the derivation of TECs from human pluripotent cells. First, they could be used to tackle the problem of T-cell reconstitution after hematopoietic stem-cell transplantation (HSCT), the only curative therapy for many hematological malignancies (Figure 1a). Before and during adolescence, the thymus begins to involute, and the production of naïve T cells decreases. This physiologic atrophy is exacerbated by conditioning regimens used before translplant and by development of graft - versus - host disease after transplant [12-14]. Whereas post-transplant reconstitution of most hematopoietic lineages is relatively swift, T-cell reconstitution is delayed up to several years in adult recipients. Even if absolute T-cell numbers recover, the full T-cell repertoire is rarely restored, which leads to increased probability of relapse, chronic viral infection, secondary malignancy and vaccine failure [12,13]. Interventions to preserve thymic integrity would allow for more robust T-cell recovery [12]. This becomes an increasingly important issue as the average age of transplant patients increases [12,14]. Current approaches in clinical trials include administration of keratinocyte growth factor (KGF), interleukin-7, growth hormone, and chemical castration using gonadotropin-releasing hormone analogues [12]. All have some clinical benefit but sometimes severe side effects. Co-transplanting patient-specific functional thymic tissue would almost certainly result in long-term benefit and enhanced survival.

A second potential reason is the attenuation of immunological aging (Figure 1b). It is widely hypothesized that improving thymic function in the older person will increase health, and perhaps extend life span [15-24]. Some rather old reports suggest that transplantation of multiple thymi into aged animals is beneficial for immune function, and increases mean, but not maximal life span, although there is evidence for significant mouse strain-dependent variation [22-24].

Congenital diseases in which a thymus is lacking are good candidates for thymus replacement therapy (Figure 1c). In these diseases, correction of or functional compensation for the genetic defect during *in vitro *differentiation may allow transplantation of autologous, iPS-derived thymic tissue (Figure 1c). Loss of *FOXN1*, an essential gene for thymic development, causes the rare 'nude/severe combined immune deficiency (SCID)' syndrome in which patients have no hair or thymus [25]. In DiGeorge syndrome (DGS), the most frequently diagnosed microdeletion in humans (1 in 4000 people), a region of chromosome 22 is monoallelically deleted. This region includes a crucial transcription factor required for the early stages of thymic development, *TBX1*. The syndrome (DGS/velofaciocardial syndrome (VFCS)) is characterized by absent or hypoplastic thymus and parathyroids, in addition to a variety of cardiac and facial malformations [26,27]. In both syndromes, allogeneic thymic transplantation has been beneficial [28-30]. Obvious problems include the allogeneic nature of the graft and the scarcity of donors. In the particular case of thymus transplantation, this problem is exacerbated by the fact that thymi from donors that would otherwise be considered young have already undergone severe age-related thymic atrophy.

A final clinical rationale is the treatment of autoimmune disease (Figure 1d). TECs derived from patient-specific iPS cells could be genetically modified to influence the process of positive and negative selection in the engrafted thymic tissue. For example, expression of a culprit autoantigen in the putative medullary cells of the graft might induce tolerance to these antigens by mediating negative selection of the immature T cells recognizing these antigens [6].

TECs derived from ES or iPS cells could also improve humanized mouse models (Figure 1e). A major challenge in immunology is the establishment of mouse models of the human immune system. Currently the best models are immunodeficient *Rag1^-/- ^ilr2g^-/- ^*or non-obese diabetic (NOD)-SCID*ilr2g^-/- ^*mice engrafted neonatally with human cord-blood hematopoietic stem and progenitor cells. In such mice, all major hematopoietic lineages are reconstituted, and even the structure of secondary lymphoid organs seems 'human'. However, human T-cell responses are weak except for alloreactivity, and peripheral T-cell homeostasis is abnormal [31-34]. An improvement of this model with more robust T-cell activity is the bone marrow/liver/thymus (BLT) mouse [35,36]. This is a NOD-SCID*ilr2g^-/- ^*mouse transplanted with a human fetal thymus and liver under the kidney capsule, and subsequently transplanted with fetal liver CD34+ progenitor cells. In these mice, both innate and major histocompatibility complex I and II-restricted, T-cell-dependent immune responses were seen. Interestingly, all T-cell development occurred in the grafted human thymus, not in the endogenous mouse thymus. These data suggest that the presence of human thymic tissue may be crucial to develop a humanized mouse. It follows that as TECs are the essential functional component of the thymus, ES or iPS-derived TECs could also be used to construct an improved humanized mouse model. In addition, by using patient-specific iPS cells, it is possible to capture in a mouse model some of the genetic diversity in disease susceptibility and immune responses among humans. Finally, iPS technology will allow cotransplantion of syngeneic human tissues (Figure 1e). In such a mouse, organ or tissue-specific immune responses in the context of autoimmunity or infection can be studied, and vaccines can be tested. The development of such a mouse may revolutionize translational research.

### Generating pluripotent stem cell-derived TECs

To date, it has not been possible to generate TECs from human ES or iPS cells, but important steps towards this goal have been made. TECs are derived from the anterior region of the third pharyngeal pouch, and are therefore of endodermal origin [5]. Subsequently, the tissue descends to the precordial region. The presumptive thymic epithelium seems largely uniform in the mouse until E12. How the different subtypes of TECs are generated is unclear, but it is well established that the E12 thymic anlage contains cells that can clonally generate both cTECs and mTECs. As fetal TEC progenitors can reconstitute a fully functional thymus [8-11], directed differentiation of ES/iPS cells to an early fetal developmental stage might suffice. This goal of generating an early fetal progenitor stands in sharp contrast to efforts to generate other more mature cell types, such a cardiomyocytes, hepatocytes and pancreatic islet cells [1,4].

The success thus far in quantitative differentiation of ES cells into pancreatic β cells, cardiomyocytes, neurons and other lineages relies on mimicking the sequential developmental states *in vivo *in ES cells *in vitro *[1,4,37-39]. As developmental cell states are induced by morphogen gradients, directed differentiation relies on the application of these same state-specific morphogens to ES cultures. Surprisingly, a recent report claimed generation of TECs from mouse ES cells through the direct application of terminal thymic maturation signals directly to pluripotent cultures [40]. The efficiency of this protocol was not reported, nor was the presence of alternative lineages assessed, and we have not been able to reproduce this approach in human pluripotent cells. Theoretically, directed differentiation of TECs should proceed by generation of definitive endoderm, followed by patterning into pharyngeal or anterior foregut endoderm [41]. Subsequently, third pharyngeal pouch and then TEC cell fates should be specified. Although definitive endoderm has been generated [42-44], subsequent specification of this germ layer to anterior and pharyngeal fates has remained a challenge. We have recently shown that dual inhibition of transforming growth factor-β/bone morphogenetic protein signaling of human ES and iPS-derived definitive endoderm led to the quantitative generation of anterior foregut endoderm, which could subsequently differentiate into cells expressing markers of parathyroids and lung [45]. It is likely that this strategy will serve as a useful platform for the generation of tissues derived from anterior foregut endoderm, including but not limited to the thymus.

## Conclusions

While the potential clinical and scientific benefits of TECs derived from human pluripotent are great, it has not yet been possible to generate TECs from human ES or iPS cells. Given recent progress in the generation of derivatives of definitive endoderm, however, we anticipate significant progress in this area in the near future.

## Competing interests

A patent application covering some of the work mentioned was filed with the US Patent and Trade Office (USPTO) by MG and HWS, and is pending.

## Authors' contributions

MG and HWS co-wrote this manuscript. Both authors read and approved the manuscript.

## Pre-publication history

The pre-publication history for this paper can be accessed here:

http://www.biomedcentral.com/1741-7015/9/51/prepub
